# EEG functional connectivity as a Riemannian mediator: An application to malnutrition and cognition

**DOI:** 10.1002/hbm.26698

**Published:** 2024-05-10

**Authors:** Carlos Lopez Naranjo, Fuleah Abdul Razzaq, Min Li, Ying Wang, Jorge F. Bosch‐Bayard, Martin A. Lindquist, Anisleidy Gonzalez Mitjans, Ronaldo Garcia, Arielle G. Rabinowitz, Simon G. Anderson, Giuseppe A. Chiarenza, Ana Calzada‐Reyes, Trinidad Virues‐Alba, Janina R. Galler, Ludovico Minati, Maria L. Bringas Vega, Pedro A. Valdes‐Sosa

**Affiliations:** ^1^ The Clinical Hospital of Chengdu Brain Science Institute, MOE Key Lab for Neuroinformation, School of Life Science and Technology University of Electronic Science and Technology of China Chengdu China; ^2^ Hangzhou Dianzi University Zhejiang Hangzhou China; ^3^ Faculty of Psychology Autonomous University of Madrid Madrid Spain; ^4^ Department of Biostatistics Johns Hopkins University Baltimore Maryland USA; ^5^ Montreal Neurological Institute‐Hospital McGill University Montreal Quebec Canada; ^6^ The George Alleyne Chronic Disease Research Centre, Caribbean Institute for Health Research University of the West Indies Cave Hill Barbados; ^7^ Centro Internazionale Disturbi di Apprendimento, Attenzione, Iperattività (CIDAAI) Milan Italy; ^8^ Cuban Center for Neuroscience La Habana Cuba; ^9^ Division of Pediatric Gastroenterology and Nutrition Massachusetts General Hospital for Children Boston Massachusetts USA; ^10^ Center for Mind/Brain Science (CIMeC) University of Trento Trento Italy

**Keywords:** causality, EEG cross‐spectrum, matrix regression, mediation analysis, Riemannian manifold

## Abstract

Mediation analysis assesses whether an exposure directly produces changes in cognitive behavior or is influenced by intermediate “mediators”. Electroencephalographic (EEG) spectral measurements have been previously used as effective mediators representing diverse aspects of brain function. However, it has been necessary to collapse EEG measures onto a single scalar using standard mediation methods. In this article, we overcome this limitation and examine EEG frequency‐resolved functional connectivity measures as a mediator using the full EEG cross‐spectral tensor (CST). Since CST samples do not exist in Euclidean space but in the Riemannian manifold of positive‐definite tensors, we transform the problem, allowing for the use of classic multivariate statistics. Toward this end, we map the data from the original manifold space to the Euclidean tangent space, eliminating redundant information to conform to a “compressed CST.” The resulting object is a matrix with rows corresponding to frequencies and columns to cross spectra between channels. We have developed a novel matrix mediation approach that leverages a nuclear norm regularization to determine the matrix‐valued regression parameters. Furthermore, we introduced a global test for the overall CST mediation and a test to determine specific channels and frequencies driving the mediation. We validated the method through simulations and applied it to our well‐studied 50+‐year Barbados Nutrition Study dataset by comparing EEGs collected in school‐age children (5–11 years) who were malnourished in the first year of life with those of healthy classmate controls. We hypothesized that the CST mediates the effect of malnutrition on cognitive performance. We can now explicitly pinpoint the frequencies (delta, theta, alpha, and beta bands) and regions (frontal, central, and occipital) in which functional connectivity was altered in previously malnourished children, an improvement to prior studies. Understanding the specific networks impacted by a history of postnatal malnutrition could pave the way for developing more targeted and personalized therapeutic interventions. Our methods offer a versatile framework applicable to mediation studies encompassing matrix and Hermitian 3D tensor mediators alongside scalar exposures and outcomes, facilitating comprehensive analyses across diverse research domains.

## Graphical abstract

This study introduces a novel mediation analysis approach using EEG cross‐spectra to investigate whether malnourishment affects children's cognitive performance. The results indicate that brain function across delta, theta, alpha, and beta band frequencies mediates the impact of malnourishment on cognitive performance in specific brain regions.

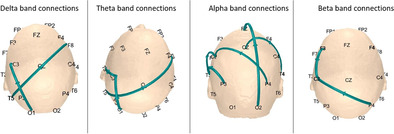

## 1 INTRODUCTION

This article extends causal mediation analysis (VanderWeele, 2016) by allowing the study of a new type of mediating variable, tensors, that has not been previously considered. The topic of causal mediation is of current interest since disentangling the relationship between environment, brain activity, behavior, and disease is crucial in neuroscience research. The need for complex entities as mediators extends from using neuroimaging, which generates more complex data types.

In recent years, there has been growing interest in addressing this type of question using statistical causal inference (Imbens & Rubin, 2015; Pearl, 2014; Rubin, 1977; Sobel, 2008). The ability to detect causal links between variables is essential, as it suggests potential interventions to modify an outcome of interest. A prime example is clinical trials where a proposed treatment's effect must be established. Mediation analysis is an essential variant of causal inference (Albert, 2008; Holland, 1988; MacKinnon et al., 2007; Preacher & Hayes, 2008; Robins & Greenland, 1992; Vanderweele, 2009) which allows one to identify intermediate steps in a causal chain of events. It is, therefore, essential for refining mechanistic explanations for causal effects.

Our concern with mediation is motivated by our research on the effects of early malnutrition on the brain, behavior, and cognitive outcomes. Childhood malnutrition affects approximately one‐fifth of children globally and leads to long‐term consequences such as increased vulnerability to inflammation and infectious diseases. While early interventions have improved survival rates, many children still experience persistent problems throughout their lives, including cognitive deficits, low IQ, poor school performance, and behavioral problems. Most studies on the long‐term cognitive and behavioral outcomes following a history of early malnutrition rely on indirect measures such as neuropsychological (Waber et al., 2014) and behavioral assessments (Galler et al., 2013) or school performance. Only a few utilize direct measures of brain function through neuroimaging (Bosch‐Bayard et al., 2022; Razzaq et al., 2023; Taboada‐Crispi et al., 2018). Detecting which long‐term effects are due to brain dysfunction might improve interventions to avoid these long‐term consequences.

Our neuroscience question was “Does the brain mediate the effects of malnutrition on later cognitive performance?” We addressed this question using Barbados Nutrition Study (BNS) data. The BNS is a unique 50+‐year longitudinal study that explores the consequences of malnutrition during infancy on brain and behavioral outcomes throughout the individual's lifespan. A cohort of children who had Protein Energy Malnutrition (PEM) limited to the first year of life and a matched control group were followed for more than 50 years. We sought to determine whether the electroencephalographic (EEG) cross‐spectral tensor (as a proxy for brain function) was a mediator and to pinpoint at which frequencies and electrodes of the EEG this effect occurred.

**FIGURE 1:**
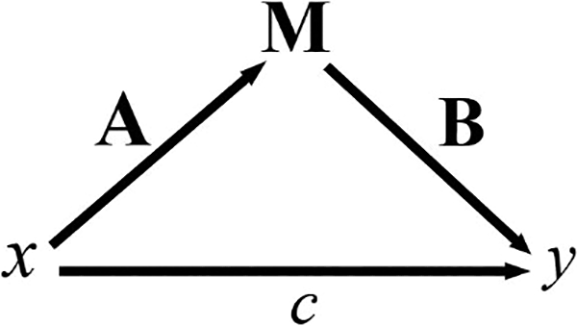
Main variables in a Mediation Analysis. The independent variable (x) might influence the dependent variable (y), either directly or through the mediator (M). Here, A, B and c are path variables describing the relationships between the variables.

To explain our objectives with more technical detail, we illustrate basic concepts involved in mediation analysis with the path diagram shown in Figure 1. Examining the causal effect of a variable x measuring exposure (also known as a treatment or intervention) on an outcome variable y means measuring the existence of path c, known as the *direct effect*. We are also interested in investigating the role of intermediate variables (mediators M) that might explain all or part of the direct effect. In the figure, the *indirect effect* of x on y is shown by the path from x to M (with a strength denoted by the variable A) and the subsequent path from M to y (with a strength represented by the variable B). Mediation analysis, therefore, must determine if A and B are different from zero.

In what follows, we assume that x, y and c are real‐valued scalar variables. M, A, and B can be different types of mathematical objects. Our interest is when M represents neuroimage‐based measures used as a proxy of brain function as a mediator of different outcomes related to IQ at 5–11 years of age. The two main measures used to date are based on functional magnetic resonance imaging (fMRI) or the EEG.

fMRI is based on dynamic changes in the magnetic susceptibility of blood (Bandettini, 2020). It has a relatively high spatial resolution but low temporal resolution and high cost. Important mediation studies based on fMRI measures have become frequent (Coan & Allen, 2004; Fleming et al., 2018; Pedroni et al., 2017). However, fMRI does not directly measure neural activity; it does so through neurovascular coupling, which poses a confounder. On the other hand, the EEG is a neuroimaging modality that offers a more direct window into the brain's electrical activity (Niedermeyer & da Silva, 2005) with a high temporal resolution albeit low spatial resolution and ill‐posed inverse problems when determining the brain activity sources. This is the imaging modality on which we focus in this paper. To date, studies using EEG measures as a mediator are uncommon. One example is earlier work from our group, which demonstrated that EEG (as a proxy for brain function) mediates the effect of a drug on cognition in Parkinson's disease patients (Bringas Vega et al., 2022). Another study from our group showed that early malnutrition was associated with accelerated cognitive decline in middle adulthood, an outcome mediated by brain state at 5–11 years of age measured using EEG (Razzaq, 2021).

An essential methodological consideration in these fMRI and EEG mediation studies is the nature of the mediator variable M, which has become increasingly complex as the biomarkers have become more elaborate. Further extending the type of biomarkers to be used is one of the aspects of our paper, and Table 1 summarizes these types of mediators. We also give some examples of their use. Briefly, the following four possibilities for the cases have been explored:

1. *Scalar* M: This is the most commonly published type of biomarker for which most procedures have been developed. Despite its widespread use and common acceptance, it poses limitations for neuroscience studies since the mediator variable, often represented as a multi‐dimensional object, needs to be resumed to a scalar. This oversimplification can produce a significant loss of information when analyzing brain biomarkers.
2. *Vector* M: a possible solution to the oversimplification in Case 1 is representing the multiple mediators as a vector. A challenge to this approach appears when the number of elements of the vector is greater than the number of subjects in the study, a phenomenon best known as high dimensional mediation.
3. M as a *Function*: as an extension of Case 2, a function can be considered a vector with infinite elements. This approach allows the mediator's effects on the outcome to be studied to vary continuously and perhaps smoothly over space or time. This differs from the standard mediation techniques that only apply if the mediating variable is summarized as a single univariate (Case 1) or vector (Case 2) variable. Note that in the vector approach, there is no inherent geometrical notion of continuity or smoothness; elements of a vector may be permuted arbitrarily without affecting the results.
4. M as a *Matrix*: fMRI and EEG connectivity mediators that vary over more than two dimensions, say space and time, are generally represented by a matrix. Some papers start with a connectivity matrix, which is then transformed into a vector (Case 2), perhaps with some dimensionality reduction technique. When recurring to vectorization of the matrix, the difference between the geometrical structure of rows and columns is ignored. To our knowledge, the entire matrix mediator case, in which this geometric structure is conserved, has not been considered.

**TABLE 1:** Types and examples of mediators.

Type of mediator	Citation example	Exposure $\left(x\in \mathbb{R}\right)$	Outcome $\left(y\in \mathbb{R}\right)$	Mediator	Summarized mediator domain
Scalar $\left(M\in \mathbb{R}\right)$	(Pathania et al., 2022)	Age‐related changes	Cognition	Resting EEG spectral slopes	Scalar $\left(M\in \mathbb{R}\right)$
Vector $\left(M\in {\mathbb{R}}^{d}\right)$	(Zhao & Luo, 2022)	Visual stimulation	Probabilistic classification learning	fMRI single trial activation from brain Regions of Interest	Vector $\left(M\in {\mathbb{R}}^{d}\right)$
Function $\left(M\left(t\right)|t\in \left[0,1\right]\right)$	(Lindquist, 2012)	Thermal stimulations	Self‐reported pain	fMRI brain activation in the right anterior insula	Function $\left(M\left(t\right)|t\in \left[0,1\right]\right)$
Matrix $\left(M\in {\mathbb{R}}^{d_{1},d_{2}}\right)$	(Nath et al., 2023)	Fluid intelligence	Working memory accuracy	Resting‐state brain connectivity.	Vector $\left(M\in {\mathbb{R}}^{d}\right)$
	(Bringas Vega et al., 2022)	Dosage of NeuroEpo	Cognition	Quantitative EEG spectral matrix	Scalar $\left(M\in \mathbb{R}\right)$
Tensor $\left(M\in {\mathbb{C}}^{d_{1},d_{1},d_{2}}\right)$	Present article	Malnutrition	Cognition	EEG cross‐spectra	Matrix $\left(M\in {\mathbb{C}}^{d_{1},d_{2}\left(d_{2}+1\right)/2}\right)$

One extra situation requires consideration.

5 M as a *Tensor*: If the biomarkers vary over more than two dimensions, say space, time, and frequency, then M is best represented as a tensor. An example we discuss below is the EEG cross‐spectral tensor (CST) formed by a frequency‐resolved collection of cross‐spectral matrices (three‐dimensions). Also, to the best of our knowledge, the tensor case has not been studied before.

Developing mediation analysis for matrix (Case 4) and tensor mediators (Case 5) is the main contribution of this article. We present, for the first time, analyses of matrix mediators. For matrix mediation, we propose the nuclear norm regularization. With this framework, we can then deal with tensor mediators by reducing them to the matrix space.

The tensor mediator we contemplate here extends from our study of the electroencephalogram (EEG) as a mediator. In this neuroimaging modality, voltage differences between multiple electrodes placed on the scalp are amplified and recorded as time series to measure the brain activity. The most widely used way of summarizing these time series is using frequency domain methods (Brillinger, 2001) based on the spectrum or cross‐spectrum. The cross‐spectrum at each frequency is a Hermitian covariance matrix of the complex Fourier coefficients; therefore, it is a generalization of the symmetric real‐valued covariance matrices of ordinary multivariate statistics to Hermitian complex‐valued covariance. The CST is formed by stacking the cross‐spectral matrices for different frequencies along a third dimension. This object has dimensions: number of EEG electrodes for the first two dimensions and frequencies for the third.

The cross‐spectral matrices we consider in this article cannot be dealt with using ordinary multivariate statistics valid for data lying in an Euclidean space. They are Hermitian and restricted to the Positive Definite cone, a curved space known as a Riemannian Manifold (Lee, 1997). A Riemannian manifold is a topological space resembling Euclidean space at each point in a local sense. When this manifold is further endowed with a differentiable structure, allowing for calculus to be performed, it is known as a differentiable manifold. A Riemannian manifold is a specialized version of a differentiable manifold, supplemented with a Riemannian metric, which introduces geometric concepts such as distance and curvature.

Leveraging the theory of Riemannian Manifolds, our group formulated a normative multivariate regression analysis by mapping the cross‐spectral matrices and the CST to the Riemannian tangent space (Li et al., 2022). Here, we extend the same general approach to tackle CST mediators.

This article is organized as follows: in Section 2, we introduce a method that transforms an EEG Riemannian CST into a Euclidean matrix representing the compressed CST, modifying a cloud of points originally in a PSD space to be dealt with as locally Euclidean when analyzing EEG data. This enables us to analyze the complete cross‐spectrum at all frequencies. The transformation involves applying a logarithmic mapping of the manifold of PSD matrices to the tangent space and using Riemannian vectorization for each frequency. The resulting matrices capture the mediating variable as a general complex‐valued matrix. We utilize a complex‐valued Matrix Regression with nuclear norm regularization to estimate mediation effects and conduct significance tests using nonparametric bootstrap and Rayleigh probability distributions. Additionally, we propose a global test for mediation based on a tensor contraction operation. In Section 3, the validity and performance of the methods are demonstrated through simulations.

To test our methods, in Section 4, we employed our well‐studied BNS dataset (Taboada‐Crispi et al., 2018) that evaluates the relationship between protein energy malnutrition (suffered only in the first year of life) and school‐age cognitive impairment (Galler et al., 2021). Rather than limiting our attention to a scalar summary of EEG spectra, we could delve into the mediation information in the EEG cross‐spectrum and decompose these effects into frequency and connectivity‐resolved components. Finally, in Section 5, we present the discussion.

## 2 MATERIALS AND METHODS

### 2.1 Mediation problem to be studied: Malnutrition and BNS

#### 2.1.1 Study site

This study was conducted in Barbados, a Caribbean country whose population is approximately 280,000 at present. The demographic makeup is 92% African/Caribbean origin, 4% Caucasian, and 4% individuals of Asian, Lebanese, and Syrian descent. In 1970, the infant mortality rate was 46 per 1000 live births. Today, it stands at 9.1, and Barbados is ranked 62/193 countries on the Human Development Index (UNDP Human Development Reports, 2023–2024). Whereas moderate–severe cases of infant malnutrition were common when this study was undertaken in the 1970s, infant malnutrition is now virtually eliminated from the island due to its improved economy and the impact of island‐wide nutrition intervention and education (Galler et al., 2021).

#### 2.1.2 BNS sample population

The selection of study participants has been described in detail (Galler et al., 1983; Taboada‐Crispi et al., 2018). Briefly, the study participants were a cohort of children born between 1967 and 1972 and diagnosed with moderate–severe protein‐energy malnutrition (PEM) restricted to the first year of life (*n* = 129, 52 females, 77 males). Inclusion criteria were as follows: birth weight > 2500 g, Apgar score >8 at birth, no pre or prenatal complications, and no encephalopathic events in childhood (See Taboada‐Crispi et al., 2018). The cohort also included a control group of healthy classmates of the PEM children who were matched by age, sex, and handedness met the same inclusion criteria but did not have histories of malnutrition (*n* = 129, 52 females, 77 males). All children in this study were enrolled in a national program that provided subsidized foods, maternal nutrition education, regular home visits by community health sisters, a preschool nursery, and routine medical care until 12 years of age. Thus, we ensured no children experienced further malnutrition or growth failure episodes.

The parents of each participant gave their informed consent at the start of the study, which was conducted under Protocol E1962 and received approval from the Institutional Review Board of Boston University Medical Center and the Ethics Committee of the Barbados Ministry of Health. Ongoing supervision of the study is under the purview of the Massachusetts General Brigham IRB (Protocol No. 2015P000329, valid until 1/19/25), and the Barbados Ministry of Health and Wellness.

#### 2.1.3 Cognitive outcome variable

The outcome variable in this study was the Wechsler Intelligence Scale for Children (WISC) (Galler et al., 1983), administered to each child when they were 5–11 years of age (at the same time as the EEG was administered). Its items were modified to incorporate local exemplars to ensure cultural relevance for Barbadian children. Additionally, a pilot trial identified specific subtests that were suitable for assessment. Verbal IQ was assessed using the following subtests: arithmetic, similarities, and digit span. Performance IQ was determined through picture completion, block design, and coding subtests. The test administration adhered to standard protocols and procedures and was conducted by a trained psychologist. The test administrator remained unaware of the children's nutritional history during the assessment.

#### 2.1.4 EEG recordings

The EEG recording procedures have been described in detail elsewhere (Taboada‐Crispi et al., 2018). The study follows standard protocols developed jointly by the Brain Research Laboratories of NYU and the Cuban Neuroscience Center that are consistent with the guidelines for resting‐state EEG analysis from the IFCN (International Federation of Clinical Neurophysiology IFCN) (Babiloni et al., 2020). The system used was a custom‐designed digital electrophysiological data acquisition and analysis system (DEDAAS) provided by the NYU Brain Research Labs (Thatcher & John, 1977). Its technical specs were: 24 channels, gain in the order of 10^4^, sharp 60‐Hz (notch‐filter), low impedance controlled to be 10 MΩ, 12‐bit analog‐to‐digital converter, sampling rate 100 Hz, 10 bits, and an antialiasing low‐pass filter (LPF) with a cut‐off frequency at 25 Hz.

Trained neurophysiologists conducted the data collection procedures and evaluated and visually inspected the EEG recordings. They were blind to the child's nutritional history. All participants were unmedicated and seated on a reclining chair in a quiet, air‐conditioned room. Each participant underwent resting‐state EEG for 8–10 min, keeping their eyes closed and not falling asleep. At the end of the recording process, individuals were asked whether they were awake throughout the session. The placement of surface electrodes was at these 19 sites: Fp1, Fp2, Fz, F3, F4, F7, F8, Cz, C3, C4, T3, T4, T5, T6, Pz, P3, P4, O1, and O2, according to the international 10–20 system, referenced to linked earlobes with a forehead ground electrode.

The data on the full cohort of *N* = 258 children were recorded in 1977–1978, when the children were 5–11 years old, stored at the NYU Brain Lab. and made available to us (courtesy of Dr Leslie Prichep). In 2016, data was recovered data from a total of 137 subjects, of which 108 records were determined to be usable after the data were processed as follows.

Computer algorithms automatically rejected data contaminated by eye or body movement or by high electrode impedance. Specifically, the AAR plug‐in from the EEGLAB 13.6.5b toolbox (Gomez‐Herrero et al., 2006) was used to eliminate ocular and muscular artifacts, mainly those in the frontal leads, Fp1 and Fp2. Furthermore, EEG epochs of 137 samples artifact‐free were selected by visual inspection of two expert neurophysiologists from the Cuban Neuroscience Center, who also carried out additional quality control for epochs with evidence of somnolence or hereto undetected artifacts or transient abnormalities.

Finally, an average reference was applied to the EEG data. This is necessary due to how the EEG signals are measured and how, in the forward model, the sources project to the channels. It is also essential to avoid the errors in the projections that can appear if we take, for example, the reference of only one of the channels. This operation is equivalent to taking an average reference in the forward model and subtracting the average error from all channels.

In order to apply an average reference to the cross‐spectral data obtained from the EEG, it is necessary to perform pre‐ and post‐multiplication by matrix $H=I-11^{T}/n_{\mathrm{ch}}$ (S. Hu et al., 2019). Here I is the identity matrix, $n_{\mathrm{ch}}$ is the number of channels, and 1 a column vector with all the components equal to one. As a result of this procedure, the matrices become rank deficient, and there is a need to delete one row and one column of the processed new matrix.

### 2.2 Construction of the EEG compressed CST

#### 2.2.1 Construction of the EEG CST from time domain signals

**FIGURE 2:**
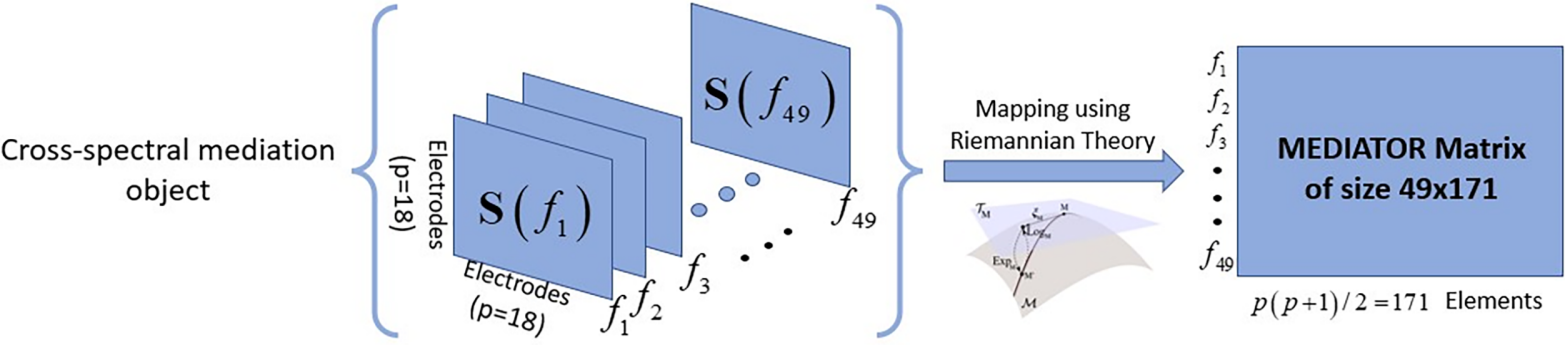
Illustration of the data transformation. Starting with a 3D object, after performing Riemannian Geometry a 2D object containing all the frequencies for each subject is obtained.

We converted the time‐domain scalp EEG signals to frequency domain signals using the Fast Fourier Transform (FFT). The EEG signal $v_{i,e,c}\left(t\right)$ (for the subject i, $i=1,\ldots ,n_{i}$, at the channel c, $c=1,\ldots ,n_{c}$ and epoch e, $e=1,\ldots ,n_{e}$) can be transformed to the frequency domain, $v_{i,e,c}\left(f\right)$ (frequency $f=\Delta f,\ldots ,n_{f}\Delta f$, where Δf is the frequency resolution) which is the complex‐value coefficients. The covariance matrix across all epochs $v_{i,e,c}\left(f\right)$ is the cross‐spectral matrix $S_{i}\left(f\right)$ at frequency f. $S_{i}\left(f\right)$ is Hermitian with elements $s_{i,c,c'}\left(f\right)$, and is formally defined as $S_{i}\left(f\right)=\frac{1}{n_{e}-1}\sum_{e}^{n_{e}}v_{i,e,c}\left(f\right)v{'}_{i,e,c}\left(f\right)$. Note that the set of all $S_{i}\left(f\right)$ for all frequencies $f=\Delta f,\ldots ,n_{f}\Delta f$ for a given subject i is a 3‐mode multi‐dimensional array with dimensions $n_{c},n_{c},n_{f}$. We call this three‐dimensional array a tensor, which generalizes the concept of a matrix and can be thought of as a collection of “slide boxes”, in which each “slide” is a cross‐spectral matrix (see left side of Figure 2). See appendix A of Li et al. (2022) for a comprehensive discussion on the cross spectra as a tensor.

In summary, the EEG cross‐spectral data is formed for each subject using n frequencies. A Hermitian matrix represents each of these frequencies. Thus, the data of every subject is represented by a three‐dimensional array (CST), where the first two dimensions correspond to each individual matrix. The third dimension represents the collection of these matrices over frequencies.

EEG data often requires regularization due to the conversion from manifold space to Euclidean space. Here, we employ the Hilbert–Schmidt (HS) method (Schneider‐Luftman & Walden, 2015) for its nonparametric, theoretically sound approach to ensuring positive definite matrices, especially beneficial for poorly conditioned data. Appendix A details the HS method application.

#### 2.2.2 Mapping from the Riemannian manifold to the Euclidean space

As mentioned above, the cross‐spectral matrices do not belong to the Euclidean space but to the manifold of positive definite (PD) matrices. They are also contained in a hyper‐dimensional cone (Barachant et al., 2013; Pennec et al., 2006). Because of this cone's curvature, using Euclidean distances for this kind of data is not feasible. Each point on the manifold is associated with a vector space known as a tangent space, and each tangent space possesses a smoothly varying inner product referred to as the Riemannian metric. This inner product within the tangent space enables us to establish Riemannian operations for analyzing data on the manifold and making inferences. These operations include the logarithm and exponential maps and the computation of Riemannian distances. (Barachant et al., 2012; Congedo et al., 2017; Pennec et al., 2006). Considering this, we use two operators to pass from the manifold to the tangent space and back. The first operator is logarithmic, which carries objects from a manifold to the tangent space and is defined as:

$${\mathrm{Log}}_{C}\left(M_{i}\right)=Q_{i}=C^{1/2}\text{logm}\left(C^{-1/2}M_{i}C^{-1/2}\right)C^{1/2},$$

where logm denotes matrix logarithm. The second and inverse to the logarithmic operator is the exponential and is defined as:

$${\mathrm{Exp}}_{C}\left(Q_{i}\right)=M_{i}=C^{1/2}\text{expm}\left(C^{-1/2}Q_{i}C^{-1/2}\right)C^{1/2},$$

were expm denotes matrix exponential.

The selection of the matrix C depends on the distance definition between two matrices in the manifold space of PD matrices. A manifold can be equipped with various Riemannian metrics. Among these metrics, the prevailing consensus in the literature is to utilize the *Affine Invariant metric* due to its property of congruence invariance, which holds significant applications in fields such as machine learning, neuroscience, and brain‐computer interfaces (Sabbagh et al., 2020). Therefore, we have chosen to employ this metric to define the Riemannian metric that will be used to construct the mediator in our method

$$d_{\mathrm{AI}}\left(A,B\right)={\left\|\text{logm}\left(A^{-1/2}{\mathrm{BA}}^{-1/2}\right)\right\|}_{F},$$

here ${\left\|\cdot \right\|}_{F}$ refer to the Frobenius norm. Based on this distance, we can calculate the mean of the manifold. It is widely agreed that the optimal mean is the Karcher mean (Karcher, 1977):

$$C={\text{mean}}_{d_{\mathrm{AI}}}\left(M_{1},\ldots ,M_{N}\right)=\arg\min_{M\in Μ}\sum_{i=1}^{N}d_{\mathrm{AI}}{\left(M_{i},M\right)}^{2}.$$

This mean is also known as geometric and Riemannian means (Bhatia, 2013; Moakher, 2005).

#### 2.2.3 Riemannian vectorization

As a result of the mapping using Riemannian geometry theory, Euclidean calculations are possible in the tangent space. Using the “Riemannian vectorization” operator (Congedo et al., 2017; Pennec et al., 2006; Sabbagh et al., 2020), we can vectorize, ensuring that the information present on the topology of the manifold is preserved. We can take advantage of the symmetric nature of our data by considering only one of the triangular parts of the matrices.

The resulting vectors representing each frequency will have a dimension of $p\left(p+1\right)/2$, where p is the number of channels. The “Riemannian vectorization” is defined as:

$$v_{i}=\text{vech}\left(\text{logm}\left(C^{-1/2}M_{i}C^{-1/2}\right)\right),$$

where vech is the operation of staking the upper diagonal of the frequency matrix in the form of a vector, C is the mean point of the Manifold space, and $M_{i}$ is the point to be mapped to the Euclidean space. In summary, we start with an EEG CST corresponding to the cross‐spectra matrices and end up with an EEG compressed CST represented by a matrix after vectorization, as illustrated in Figure 2.

### 2.3 A multivariate causal mediation model with the compressed CST

Several theoretical approaches exist for mediation analysis for whatever type of mediator structures. One of the most mature is based on the use of the “potential outcomes” or “counterfactuals” notation introduced by Rubin (1974) and fully described in Imbens and Rubin (2015), which we follow in this article.

#### 2.3.1 Notation

This section defines the causal mechanisms that transmit the effect of a treatment variable on an outcome through a multivariate mediator, where the mediator is a matrix (e.g., the compressed CST). In the following, let the symbol − above a variable denote that it is a random variable (RV) (e.g., $\bar{x}$) according to Table 2. In contrast, the variable without the − symbol denotes a realization of that RV (e.g., $\bar{x}=x$). Let $\bar{x}$ indicate an exposure/treatment for a given subject (e.g., malnutrition) and $\bar{y}$ an outcome (e.g., IQ scores).

**TABLE 2:** Mathematical notation.

x∈ℂ	Scalar
$x\in {\mathbb{C}}^{p}$	Vector of size p, with $x={\left[x_{1},\ldots ,x_{p}\right]}^{T}$
$X\in {\mathbb{C}}^{p\times q}$	Matrix of size p×q
$\bar{x}$	Scalar random variable
$\bar{X}$	Matrix random variable

First, we formalize the notion of a causal treatment effect. Assume that the exposure ${\bar{x}}_{i}$, for individual $i=\left(1,\ldots ,n\right)$, is binary has two exposure levels, ${\bar{x}}_{i}=1$(e.g., subjects who suffered from malnutrition) and ${\bar{x}}_{i}=0$ (e.g., control subjects). Then, the possible outcomes for individual i for one observation in the treatment condition can be generally denoted as ${\bar{y}}_{i}\left({\bar{x}}_{i}\right)$, specifically ${\bar{y}}_{i}\left(1\right)$ for the malnutrition outcome and ${\bar{y}}_{i}\left(0\right)$ for the control outcome. Ideally, we could observe the individual under both treatments, and $\bar{y}\left(1\right)-\bar{y}\left(0\right)$ would be the unit (or individual) treatment effect indicating the changes or effects that occur due to malnutrition. In practice, we can observe only one of the possible outcomes since an individual cannot be malnourished and nourished in the same time. The unobservable treatment outcome is *counterfactual*. Importantly, though the unit‐level treatment effect is unobservable, the average treatment effect over a population can be estimated with the expected value $Ε\left(\bar{y}\left(1\right)-\bar{y}\left(0\right)\right)$. This quantity allows for a statistical assessment of the influence of malnutrition on the outcome, obtained with the so‐called counterfactual or potential outcomes approach adopted in this article (Robins & Greenland, 1992; Rubin, 1974).

Furthermore, suppose there is a high‐dimensional mediator, represented as a p×q dimensional matrix, in our case, a compressed EEG cross‐spectra tensor $\bar{M}$, that lies in the path between treatment and outcome.

Mediation analysis goes beyond calculating average treatment effects. Instead, it seeks to quantify the effect of a treatment that operates through a particular mechanism. Let the $\bar{M}\left(x\right)$ represent the RV value of the mediators if the exposure $\bar{x}$ is set to x. Similarly, let $\bar{y}\left(x,M\right)$ denote the RV outcome if $\bar{x}$ is set to x and $\bar{M}$ is set to M. Here we only observe one of the potential outcomes, and the observed outcome, $\bar{y}$, is $\bar{y}\left(\bar{x},\bar{M}\left(\bar{x}\right)\right)$, which depends upon both the treatment status and the level of the mediator under the observed treatment status.

#### 2.3.2 Quantities of interest

Following the definitions by Hicks and Tingley (2011), the natural direct effect (NDE) of exposure $\bar{x}$ on outcome $\bar{y}$ comparing $\bar{x}=1$ with $\bar{x}=0$ intervening to set $\bar{M}$ to what it would have been if exposure had been $\bar{x}=x$ for x=0,1 is defined as:

(1) $$\mathrm{NDE}=\bar{y}\left(1,\bar{M}\left(x\right)\right)-\bar{y}\left(0,\bar{M}\left(x\right)\right).$$

Essentially, the natural direct effect assumes that the intermediate $\bar{M}$ is set to $\bar{M}\left(x\right)$, the level it would have been for each individual had exposure been x, and then compares the direct effect of exposure. The natural indirect effect or causal mediation effect (CME) is defined as:

(2) $$\mathrm{CME}=\bar{y}\left(x,\bar{M}\left(1\right)\right)-\bar{y}\left(x,\bar{M}\left(0\right)\right).$$

The natural indirect effect assumes that exposure is set to some level $\bar{x}=x$, for x=0,1 and then compares what would have happened if the mediator were set to what it would have been if exposure had been $\bar{x}=1$ versus what would have happened if the mediator were set to what it would have been if exposure had been $\bar{x}=0$.

What should be clear is that while we observe $\bar{y}\left(x,\bar{M}\left(1\right)\right)$ for units with $\bar{x}=x$, we do not observe the counterfactual outcome $\bar{y}\left(x,\bar{M}\left(0\right)\right)$ in the typical research design with one observation per unit. This makes identifying causal mechanisms more difficult than identifying treatment effects, and requires an additional assumption known as sequential ignorability, discussed below. In practice, just as with treatment effects, we are interested in an average of the direct effect ADE, and is defined as:

(3) $$\mathrm{ADE}=Ε\left(\bar{y}\left(1,\bar{M}\left(x\right)\right)-\bar{y}\left(0,\bar{M}\left(x\right)\right)\right).$$

Similarly, the average causal mediation effect (ACME) is defined as:

(4) $$\text{ACME}=Ε\left(\bar{y}\left(x,\bar{M}\left(1\right)\right)-\bar{y}\left(x,\bar{M}\left(0\right)\right)\right).$$

#### 2.3.3 Assumptions

For these effects to be well defined, it must be hypothetically possible to intervene on the mediator without affecting the exposure. We also assume no interaction between treatment and mediator throughout this article. No interaction corresponds to effect homogeneity such that causal effects are constant across cases. If there is cause–mediator interaction, the mediator is also a moderator that changes the effect of $\bar{x}$ on $\bar{y}$, either attenuating or amplifying it, as a function of $\bar{M}$ (Kline, 2015). In Section 5, we evaluate this assumption and others in the context of our dataset.

Because a potential outcome required for calculating indirect and direct effects is never observed, the ACME or ADE is not identified in the standard design, where the treatment is randomized/ignorable conditional pretreatment covariates, and the mediator/outcome variables are measured. An additional assumption is therefore required: sequential ignorability (SI; VanderWeele & Vansteelandt, 2013). We will use the symbol ⊥⊥ to denote statistical independence. The assumption can be written, for $x,x^{\prime }=0,1$ and $x\neq x^{\prime }$, as:

(5) $$\begin{array}{l} 1:\bar{y}\left(x,\bar{M}\left(x\right)\right)⊥⊥\bar{x}\\ 2:\bar{y}\left(x,M\right)⊥⊥\bar{M}\left|\bar{x}\right.\\ 3:\bar{M}\left(x\right)⊥⊥\bar{x}\\ 4:\bar{y}\left(x,M\right)⊥⊥\bar{M}\left(x^{\prime }\right). \end{array}$$

Together, these assumptions state that there is no confounding for the associations between (1) exposure $\bar{x}$ and outcome $\bar{y}$; (2) mediators $\bar{M}$ and outcome $\bar{y}$; (3) exposure $\bar{x}$ and mediators $\bar{M}$; as well as no confounding for the associations between mediator and outcome that is affected by the exposure (4).

#### 2.3.4 Estimation of direct and indirect effects

We will now present a regression‐based approach for estimating the direct and indirect effects. Suppose assumptions (1)–(4) hold, that $\bar{y}$ and $\bar{M}$ are continuous and that the following regression models for $\bar{y}$ and $\bar{M}$ are correctly specified:

(6) $$\begin{array}{l} E\left(\bar{M}|\bar{x}=x\right)=a_{0}+Ax,\\ E\left(\bar{y}|\bar{x}=x,\bar{M}=M\right)=b_{0}+\mathrm{cx}+\mathrm{BM}. \end{array}$$

Here A, B,and c are coefficients to be estimated. This model incorporates the assumptions of linear relationships between exposure, mediators, and outcomes and the lack of any exposure‐mediator interaction in the outcome regression.

With the above model, the average natural direct effect can be expressed as follows:

(7) $$E\left(\bar{y}\left(1,\bar{M}\left(x\right)\right)-\bar{y}\left(0,\bar{M}\left(x\right)\right)\right)=c\left(x-x^{\prime }\right)$$

Similarly, we define the “element‐wise” average indirect effect in terms of the Hadamard product as:

(8) $$E\left(\bar{Y}\left(x,\bar{M}\left(1\right)\right)-\bar{Y}\left(x,\bar{M}\left(0\right)\right)\right)=\left(x-x^{\prime }\right)A\circ B$$

and the “summarized” average indirect effect in terms tensor contraction operation $\sum_{i}^{p}\sum_{j}^{q}a_{\mathrm{ij}}b_{\mathrm{ij}}$ (Karahan et al., 2015) as:

(9) $$E\left(\bar{y}\left(x,\bar{M}\left(1\right)\right)-\bar{y}\left(x,\bar{M}\left(0\right)\right)\right)=\left(x-x^{\prime }\right)\sum_{i}^{p}\sum_{j}^{q}a_{\mathrm{ij}}b_{\mathrm{ij}}.$$

The definition in Equation (8) will be useful in finding specific mediator elements in the matrices A and B., while the one given in Equation (9) will help test for a global mediation effect.

When the counterfactuals are well‐defined and sequential ignorability assumptions in Equation (5) hold, the right‐hand sides of Equations (8) and (9) above identify causal mediation effects [for a detailed explanation, see VanderWeele (2015, p. 5)]. When one or more assumptions fail to hold, or if the counterfactuals are not well defined, the right‐hand sides may still be used in exploratory analysis to identify potential mediators. For example, they could be used to identify linear combinations of elements of the positive definite matrix corresponding to specific brain functions, suggesting mediation through correlates of those elements. In practice, it is uncertain whether these expressions should be understood as implying causation or if they are merely for exploratory purposes. This uncertainty underscores the intricate nature of causal mediation analysis and the need for a nuanced understanding of its implications. Various studies have delved into the complexities of mediation analysis, highlighting the importance of precise counterfactual definitions and the implications of assumptions in identifying causal effects (Hicks & Tingley, 2011; Imai et al., 2010; Pearl, 2014; VanderWeele, 2016). These works emphasize the significance of considering scenarios where assumptions may not hold and how exploratory analysis can offer valuable insights into potential mediators.

### 2.4 Matrix regressions required for the mediation model

Fitting the models for the observed data is straightforward if the number of mediators is small. However, for traditional mediation methods, if the number of mediators inside a matrix or tensor significantly exceeds the sample size, potential problems can be caused. In that case, it can lead to statistical inefficiency, overfitting, and estimates become unstable. Below, we create a method that overcomes this high dimensional potential problem by transforming an EEG Riemannian CST into a Euclidean cross‐spectral matrix carrying Riemannian Geometrical objects to Euclidean Geometry. This enables us to analyze the complete cross‐spectrum at all frequencies using matrix regressions.

#### 2.4.1 Nuclear Norm‐based matrix regression of the mediator on the treatment

The next step involves the quantitative determination of the mediation analysis coefficients. Since the mediator is a matrix and both exposure and response are scalars, we cannot use classical univariate regressions. Instead, we will use what we refer to as Nuclear Norm‐Based Matrix Regression (NNMR) for both the regression of the mediator on the exposure and the response on the mediator and exposure. Based on the work done by Kong et al. (2020), we first present regression where the response is a matrix, and the stimulus is a scalar. Let $\left\{\left(x_{i},M_{i}\right):1\leq i\leq n\right\}$ denote independent identically distributed (i.i.d.) observations, where $x_{i}={\left(x_{i1}\ldots ,x_{\text{is}}\right)}^{T}$ is a s×1 vector of scalar covariates and $M_{i}$ is a p×q response matrix

(10) $$M_{i}=\sum_{l=1}^{s}x_{\mathrm{il}}A_{l}+E_{i}.$$

**FIGURE 3:**
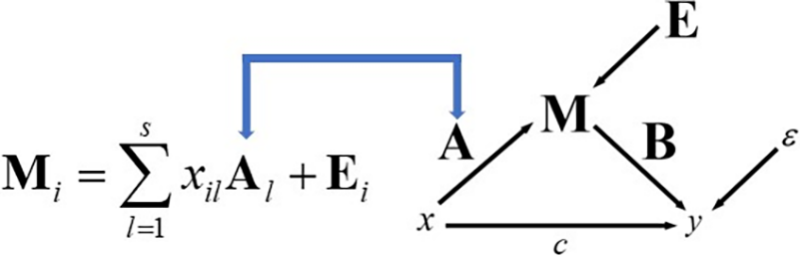
Illustration of the A regression coefficient in the mediation model.

Here $A_{l}$ is a p×q coefficient matrix characterizing the effect of the lth covariate on $M_{i}$ and $E_{i}$ is a p×q matrix of random effects with mean 0. Figure 3 shows the schematic of the model path diagram and its equation representation.

To estimate A we must solve the following minimization problem:

(11) $$\min_{A}\frac{1}{2}\sum_{i=1}^{n}{\left\|M_{i}-\sum_{l}x_{\mathrm{il}}A_{l}\right\|}_{F}^{2}+\lambda \sum_{l}{\left\|A_{l}\right\|}_{*}.$$

Here, the nuclear norm is defined as ${\left\|A\right\|}_{*}=\sum_{j}\sigma_{j}\left(A\right)$ and $\sigma_{j}\left(A\right)$ is the jth singular value of the A matrix. The parameter A is chosen using the Akaike Information Criterion (AIC; Akaike, 1974) or Bayesian Information Criterion (BIC; Schwarz, 1978) according to Equations (14) and (15).

The Nesterov algorithm (Nesterov, 2004), crucial for estimating high‐dimensional matrix data, can be used to solve Equation (11). This method is similar to the gradient descent algorithm, but with the difference that it extrapolates the previous two algorithmic iterates to generate the next search point. The process of extrapolation incurring negligible computational expense leads to a significant enhancement in the convergence rate (Nesterov, 1983).

#### 2.4.2 NNMR of the response on the mediator

For the case where the response variable is a scalar, and the predictor is a matrix, we employ the methodologies and findings established in the study conducted by Zhou and Li (2014). Let y be a scalar response, M be a matrix covariate of size p×q, and B the coefficient matrix of the same size

(12) $$y=\left\langle M,B\right\rangle +\mathrm{cx}+\varepsilon .$$

**FIGURE 4:**
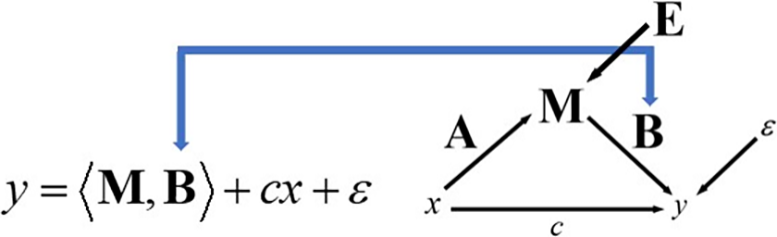
Illustration of the B regression coefficient in the mediation model.

The inner product between two matrices is defined as $\left\langle M,B\right\rangle =\mathrm{Tr}\left({\mathrm{MB}}^{H}\right)=\mathrm{vec}\left(B^{H}\right)\mathrm{vec}\left(M\right)$, where $\mathrm{vec}\left(\cdot \right)$ is the vectorization operator that stacks the columns of a matrix into a vector. Here ε is a normal error with mean zero and variance $\sigma^{2}$. An illustration showing where this coefficient is located in the path diagram can be seen in Figure 4.

To solve (12), we need to find the solution of the minimization problem

(13) $$\min_{B}\frac{1}{2}\sum_{i=1}^{n}{\left(y_{i}-{\mathrm{cx}}_{i}-\left\langle B,M_{i}\right\rangle \right)}^{2}+\lambda {\left\|B\right\|}_{*}.$$

According to Equation (13), the minimum value of B and the parameter c can be found using the Nesterov algorithm (Nesterov, 2004), as was the case for the NNMR of the mediator on the treatment described in the previous section. We used the methods in the MATLAB toolbox TensorReg (Zhou, 2017) to perform the tensor operations and estimate the B and c coefficients.

Although cross‐validation is usually chosen to obtain the penalization parameter λ, using AIC or BIC significantly improves computation time. Let $y_{i}$ denote the ith observation of $\bar{y}$, ${\hat{y}}_{i}\left(\lambda \right)$ represent the estimated response under a given parameter λ, and $v^{2}$ is the variance of the error term from equation (12). Then for this model, AIC and BIC are defined by

(14) $$\mathrm{AIC}\left(\lambda \right)=\frac{\sum_{i}{\left\{y_{i}-{\hat{y}}_{i}\left(\lambda \right)\right\}}^{2}}{v^{2}}+2\mathrm{df}\left(\lambda \right),$$

(15) $$\mathrm{BIC}\left(\lambda \right)=\frac{\sum_{i}{\left\{y_{i}-{\hat{y}}_{i}\left(\lambda \right)\right\}}^{2}}{v^{2}}+\log\left(n\right)\mathrm{df}\left(\lambda \right).$$

AIC and BIC are defined similarly for the regression presented in (10) with the subtle difference of the matrix response variable. Here, df denote degrees of freedom, and its calculation is detailed in Appendix B.

##### Investigating the instance of a complex inner product $\left\langle B,M\right\rangle$

If the mediator represents a complex cross‐spectral matrix and the resulting variable is a scalar, it is crucial to consider this while calculating the coefficient obtained from Equation (8), as the inner product will yield a complex value. To tackle this issue, we incorporated a penalization constant into the imaginary component of the inner product of B and M, effectively nullifying it and subsequently satisfying the conditions imposed by Equation (8).

Assuming that B and M are complex matrices, we have $\left\langle B,M\right\rangle =\left(\left\langle B^{R},M^{R}\right\rangle -\left\langle B^{I},M^{I}\right\rangle \right)+i\left(\left\langle B^{R},M^{I}\right\rangle +\left\langle B^{I},M^{R}\right\rangle \right)$, were the superscripts R and I account for the real and imaginary parts of the complex quantities, respectively. In matrix notation, we then can express:

$$y^{R}=\left[\begin{array}{l} B^{R}\\ B^{I} \end{array}\right]\cdot \left[\begin{array}{l} M^{R}\\ -M^{I} \end{array}\right],y^{I}=\left[\begin{array}{r} B^{R}\\ B^{I} \end{array}\right]\cdot \left[\begin{array}{l} \lambda M^{I}\\ \lambda M^{R} \end{array}\right]$$

where λ is the penalization parameter and $y^{I}=0$ since the outcome must be scalar.

### 2.5 Significance tests of mediation coefficients A, B and A∘B

To assess the significance of the estimated parameters, we follow a procedure based on the bootstrap procedure (Efron & Tibshirani, 1994).

1. Estimate the parameters A, B, and A∘B using the mediation model to be tested and the original data.
2. Generate 1000 (Preacher & Hayes, 2008) new datasets from the original data using the nonparametric bootstrap procedure and fit the mediation model. This allows us to estimate the sampling distribution of the parameters of interest.
3. The significance test is based on shifting the mean of the distributions following the details described below.
4. Compare the actual values from our data from step 1 with the tail probabilities to compute p‐values.
5. These tests are corrected for multiple comparisons using the False Discovery Rate procedure.
6. Create a $-\log_{10}$ probability map with limits from the FDR threshold set to plus $3\log_{10}$ units. These plots are shown with a heat colormap.

#### 2.5.1 Construction of the null hypothesis for particular cases

1. Using the bootstrap samples, calculate the standard deviation of the bootstrap distribution.
2. Standardize each element of the estimated matrices A and B and scalar c by dividing them by the standard deviation derived from the bootstrapped samples.
3. These standardized normal variables are real‐valued for the Riemannian spectra (diagonal of the A and B matrices) and complex‐valued for the Riemannian cross‐spectra (off‐diagonal elements of A and B).
4. Use the Rayleigh Distribution to evaluate the tail probability distributions (Abrahams & Papoulis, 1984). For the Riemannian cross‐spectra, it is well known that if the real and imaginary parts of a complex number are independently distributed with zero mean, then the absolute value of the complex number forms a Rayleigh distribution

(16) $$f\left(x,\delta \right)=\frac{x}{\delta^{2}}e^{-x^{2}/\left(2\delta^{2}\right)},\;x\geq 0,$$

where δ is the scale factor. On the other hand, its cumulative distribution function is given by

(17) $$F\left(x;\delta \right)=1-e^{-x^{2}/\left(2\delta^{2}\right)}.$$

#### 2.5.2 Multiple comparison control procedures with the false discovery rate

As mentioned, we compute the absolute value of all the elements that form each of the estimated coefficients A and B divided by the standard deviation of the bootstrap samples. We find the extreme upper tail probabilities using the cumulative distribution function. We assess the significance of the estimates using the false discovery rate (FDR; Storey, 2003) to find the threshold for which all *p* values are less than some predetermined value.

#### 2.5.3 Significance of the tensor contraction mediation effect

We aim to conduct a hypothesis test to determine whether we can reject the null hypothesis, which posits that the product of the two variables, $\sum_{i}\sum_{j}a_{\mathrm{ij}}b_{\mathrm{ij}}$, is not significantly different from zero. It is important to note that the quantity of interest is complex valued, thus we use Hotelling's T‐squared statistic as a multivariate test statistic. We treat the real and imaginary components of the complex numbers as two separate variables. Initially, we generate a collection of bootstrap samples with replacement from the original data and estimate a mediation effect for each bootstrapped dataset. Subsequently, we calculate Hotelling's T‐squared statistic $t^{2}$, accounting for the covariance matrix, and convert it to the *F*‐statistic $F=\frac{n-n_{p}}{n_{p}\left(n-1\right)}t^{2}$,where n is the number of samples and $n_{p}$ is the number of variables. Utilizing the obtained *F*‐statistic, we proceed to compute the corresponding *p*‐value associated with the mediation effect by employing the cumulative distribution function of the F‐distribution.

## 3 SIMULATIONS TO EVALUATE THE MATRIX MEDIATION ANALYSIS EFFECT

To determine the mediation effect, it is necessary to multiply the estimated parameters A and B corresponding to the indirect effect and assess its significance. We generated four sets of data with different relationships among the treatment, mediator, and outcome variables to simulate the proposed methods. For all four simulations, a sample of 100 subjects was divided randomly into equal amounts between treatment and control groups (values 0 and 1).

In Simulation 1, the outcome was set to depend only on the exposure, that is $y_{i}=x_{i}+\eta_{i}$ and $M_{i}=\varepsilon_{i}$. Here $\eta_{i}$ and $\varepsilon_{i}$ are errors with standard normal distribution. In this simulation, the effect of the mediation effect should be zero. In Simulation 2, we used the same variables as in Simulation 1. However, in this case, we only allow the mediator to depend on the exposure $M_{i}=A\left(x_{i}+\xi \right)+\varepsilon_{i}$, but the outcome does not rely on the mediator. Here, ξ is $N\left(1,0.1\right)$. In Simulation 3, the outcome depends only on the mediator $y_{i}=M_{i}+\eta_{i}$ and not on the exposure. In addition, the mediator doesn't depend on the exposure $M_{i}=A\xi_{i}+\varepsilon_{i}$. Finally, in Simulation 4, we tested the mediation effect on the outcome variable when there is an effect of x on M and M on y, respectively. For this, we used the same configuration as in Simulation 3, but with $M_{i}=A\left(x_{i}+\xi_{i}\right)+\varepsilon_{i}$.

**FIGURE 5:**
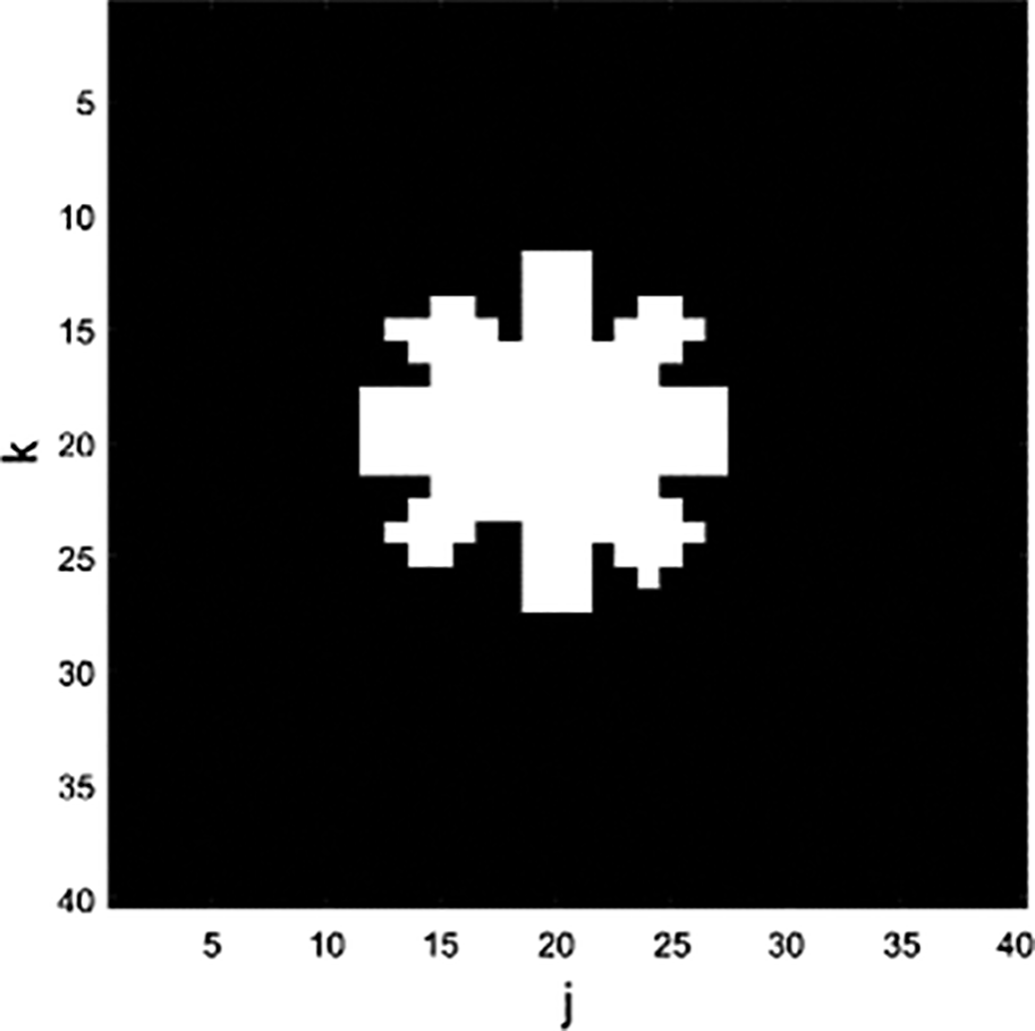
The parameter A is chosen to generate the simulation data. Here, j and k represent the number of elements of the simulation figure.

The parameter A was chosen to take the shape of an asterisk (see Figure 5), with the outer part of the matrix formed by zeros and the inner part made of the complex magnitude $\sqrt{2}+i\sqrt{2}$.

**FIGURE 6:**
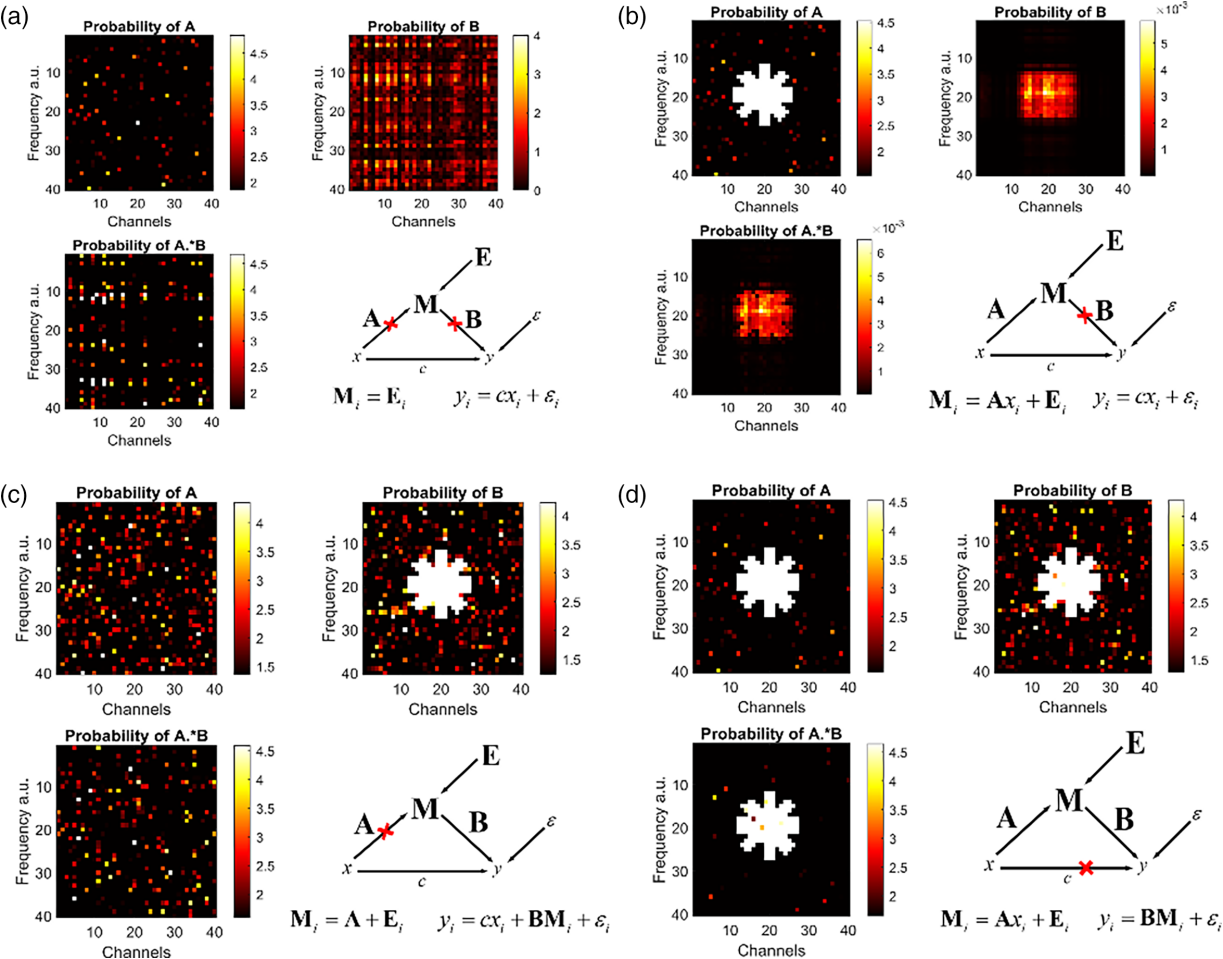
Probability of the coefficients A, B, and A∘B for the four simulations. (a) Simulation 1: Here the outcome depends only on the stimulus. (b) Simulation 2: The mediator and the outcome depend on the stimulus, but the outcome does not depend on the mediator. (c) Simulation 3: The output depends on the mediator and the treatment, but the mediator does not depend on the treatment. (d) Simulation 4: The mediator depends on the input and the outcome of the mediator.

These values allowed us to emulate the complex cross‐spectral data used in the study of Barbados. In Figure 6 the $-\log_{10}\left(p‐\text{value}\right)$ map is shown. The *p*‐values are easier to visualize in this scale as a *p*‐value of .05 corresponds to $-\log_{10}\left(0.05\right)=1.3010$.

As displayed in Figure 6a, as expected, there is no significant mediation effect since all the effects of the outcome occur through the direct effect. In this case, nothing is significant.

For Simulation 2 (Figure 6b), there is only significance in the parameter A with *p*‐values less than .0195, but the mediation effect is not significantly different from 0 as in Simulation 1.

In the case of Simulation 3, the method correctly predicts the parameter B, as can be seen in Figure 6c. The threshold *p*‐value for B was .0599. Clearly, there are a certain number of false positives. It can be explained by two factors: the sample size and low rank of the image. For the first factor, the results improved with sample sizes greater than 300. For the second, the prediction of the coefficient improves when employing the ridge regression version of the elastic net. This situation is explained in detail below.

Finally, in Simulation 4 (Figure 6d), the direct effect was forced to zero, meaning that the outcome does not depend on the stimulus directly. In this way, we were able to see the effect of the exposure only through the mediator variable. The threshold *p* values for A, B, and A∘B were, in this case, .0195, .0459, and .0183, respectively.

Due to the low rank of the A signal, the ridge version of the Elastic Net regularization was used to estimate the B parameter. The algorithm allows changing the value φ from 1 to 2, where $P\left(\varphi ,\beta \right)=\varphi {\left\|\beta \right\|}^{2}+\varphi \left\|\beta \right\|$ represents the penalty term applied to the regression. This range includes the extreme cases of Lasso regularization (φ=1) and Ridge regularization (φ=2). Lasso regularization usually fails when the variables are highly correlated. On the other hand, its performance increases when the signal is high rank. On the contrary, ridge regression performs well when the variables are highly correlated but lacks accuracy for high‐rank signals.

To find the best estimator of the regression coefficients, we searched for the λ value for which the AIC was minimum. We provided the algorithm with 30 possible lambda values ranging from 0.0067 to 148. This method is time and computationally‐consuming, especially when it is necessary to perform the bootstrap. We used the high‐performance computing resources of our lab (12 nodes with 24 cores and 128 GB of memory each). With all these resources and parallel processing, we reduced the processing time from approximately 1 h for 1000 bootstraps to 10 min. It should be noted that even when the choice of the AIC can present itself costly in terms of time and resources, it is more efficient than cross‐validation.

The algorithm searches for the λ value minimizing the AIC and BIC, which provides a compromise between model fit and model complexity. In both cases, we choose AIC over BIC since it tends to favor more complex. In addition, AIC considers Type II errors to be more undesirable than Type I errors unless n is very small (Dziak et al., 2020), which can help in this case to detect a real effect in the matrix coefficients when it exists.

### 3.1 Simulation of power curves for the mediation effect quantified by $\sum_{i}\sum_{j}a_{\mathrm{ij}}b_{\mathrm{ij}}$

**FIGURE 7:**
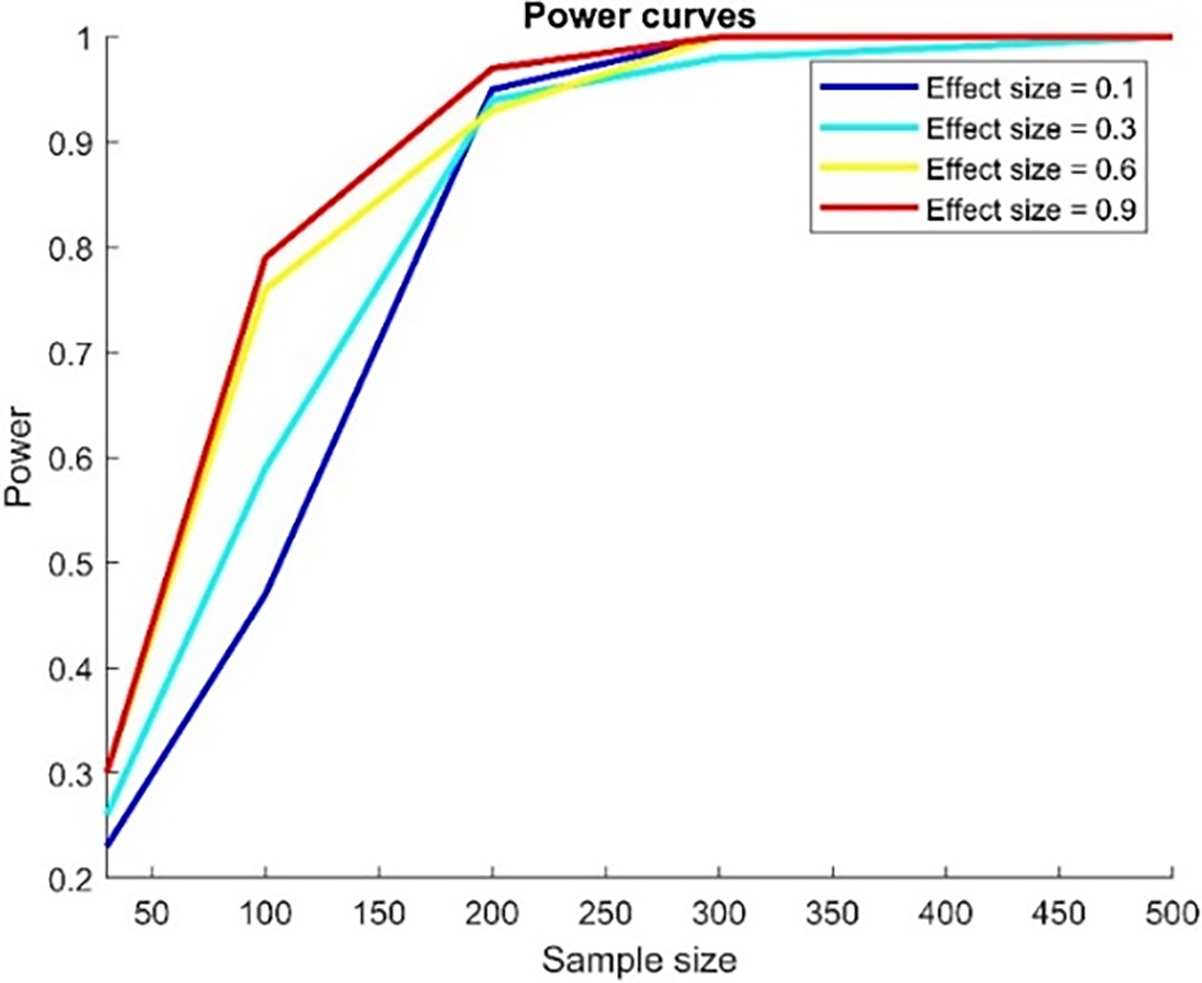
Power curves for the mediation effect $\sum_{i}\sum_{j}a_{\mathrm{ij}}b_{\mathrm{ij}}$.

We simulated the power curves to establish the requisite sample size for the study and evaluate the probability of correctly rejecting the null hypothesis when the alternative hypothesis is true. We manipulated the effect sizes from 0.1 to 0.9 and the sample sizes from 30 to 500. A graphical representation of the findings is presented in Figure 7. As visible from the figure, when the sample size is 100 or greater, the power of the test is nearly 0.8.

## 4 RESULTS

### 4.1 Statistical comparison between control and PEM groups for different frequency EEG data representations

We previously argued that Riemannian manifold mapping is a method that captures the geometric nature of SPD matrices. To argue over this point, we conducted a *t*‐test statistic between the groups of Control and PEM. In this way, we can quantify the differences between groups. We are interested in finding out how different the means are of the two sample distributions of data considering three EEG data representations. First, we evaluate the cross‐spectra representation; Second, only the spectra part is tested; and third, we try to find the differences using the Riemannian manifold mapping. MATLAB/OCTAVE provides a function to perform this operation for real numbers; however, some changes were needed to make it applicable to complex numbers. The procedure for this case is given by the methods explained in Abdullah et al. (2015).

**FIGURE 8:**
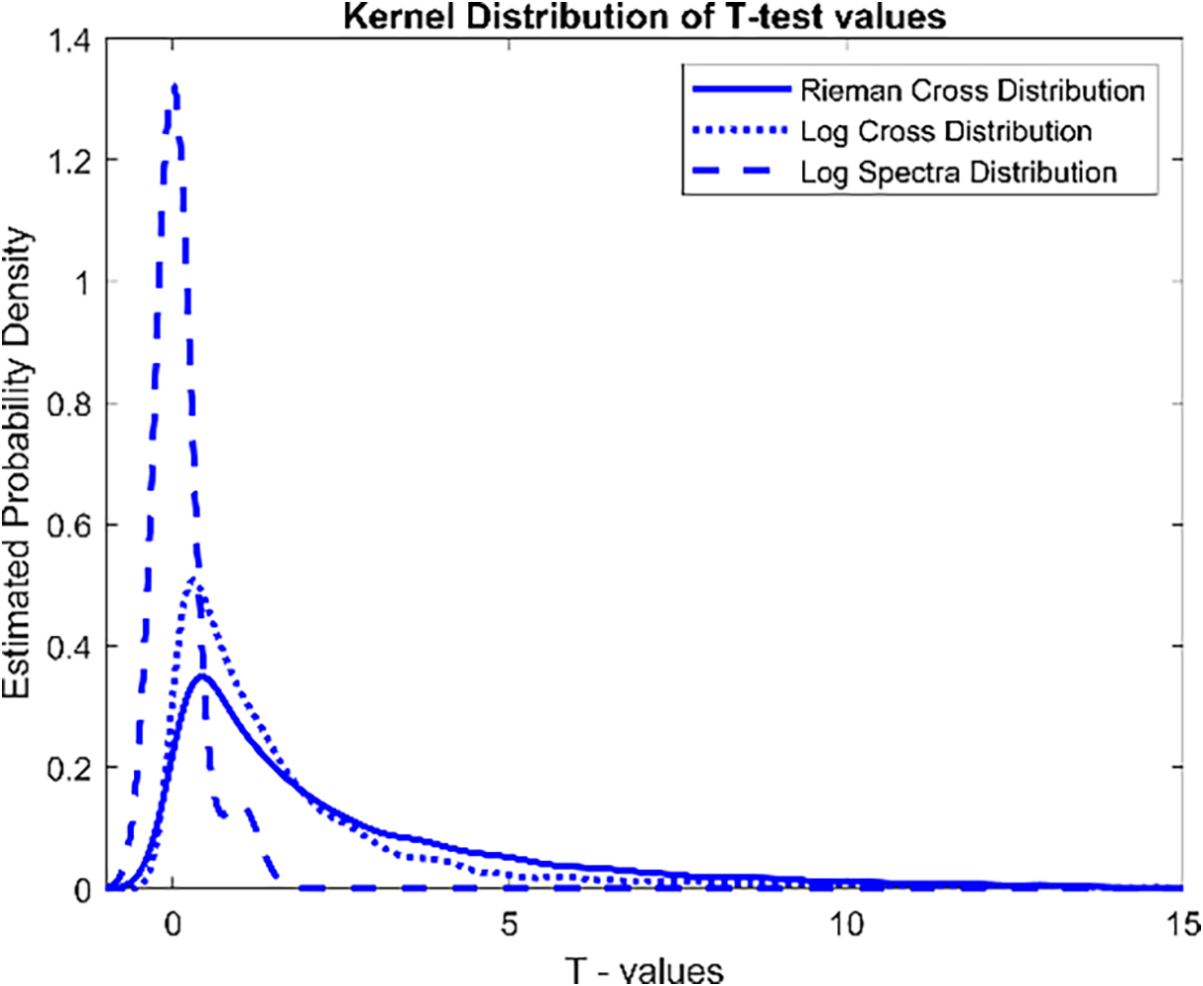
Kernel distribution of the *t* values resulting from the test.

The comparison between the *t*‐statistics for the spectra, cross‐spectra, and Riemannian manifold data of the Control and PEM groups was conducted as follows: first, we find the *t*‐statistic values. The number of *t*‐tests performed might not always be the same for the three data types under consideration. For example, if we were to consider only the spectra rather than the cross‐spectra elements of the matrices, the number of tests to perform would decrease. For the EEG cross‐spectral and Riemannian cross‐spectral data, there are 171 tests (due to the vectorization of the lower triangular part of the cross‐spectral matrices). Due to these disparities, we found a Kernel distribution of the t‐values. The Kernel distribution has the advantage of being a standardized representation distribution of the histogram since the area under the curve is one of the three distributions. Figure 8 shows the Kernel distributions for the three data representations.

As can be seen, the Riemannian representation finds more differences between the Control and PEM groups, as seen in the shift to the right and the flatness of the Kernel probability function. This shift represents greater t‐values than the other two data representations. Following the Riemannian representation, the Log Cross‐Spectra is the one that found more differences, and finally, with the least differences found is the Log Spectra Data representation. Previously, Taboada‐Crispi et al. (2018) had found differences between the two groups using only the Log‐spectra of the EEG. However, the EEG log cross‐spectra differences have not been studied before for the BNS. These more significant differences constitute a novelty and open the way to establish precisely where these differences are located in electrode locations and frequency bins.

### 4.2 The EEG Riemannian cross spectrum as a mediator between malnutrition in the first year of life and cognitive defects at school age

**FIGURE 9:**
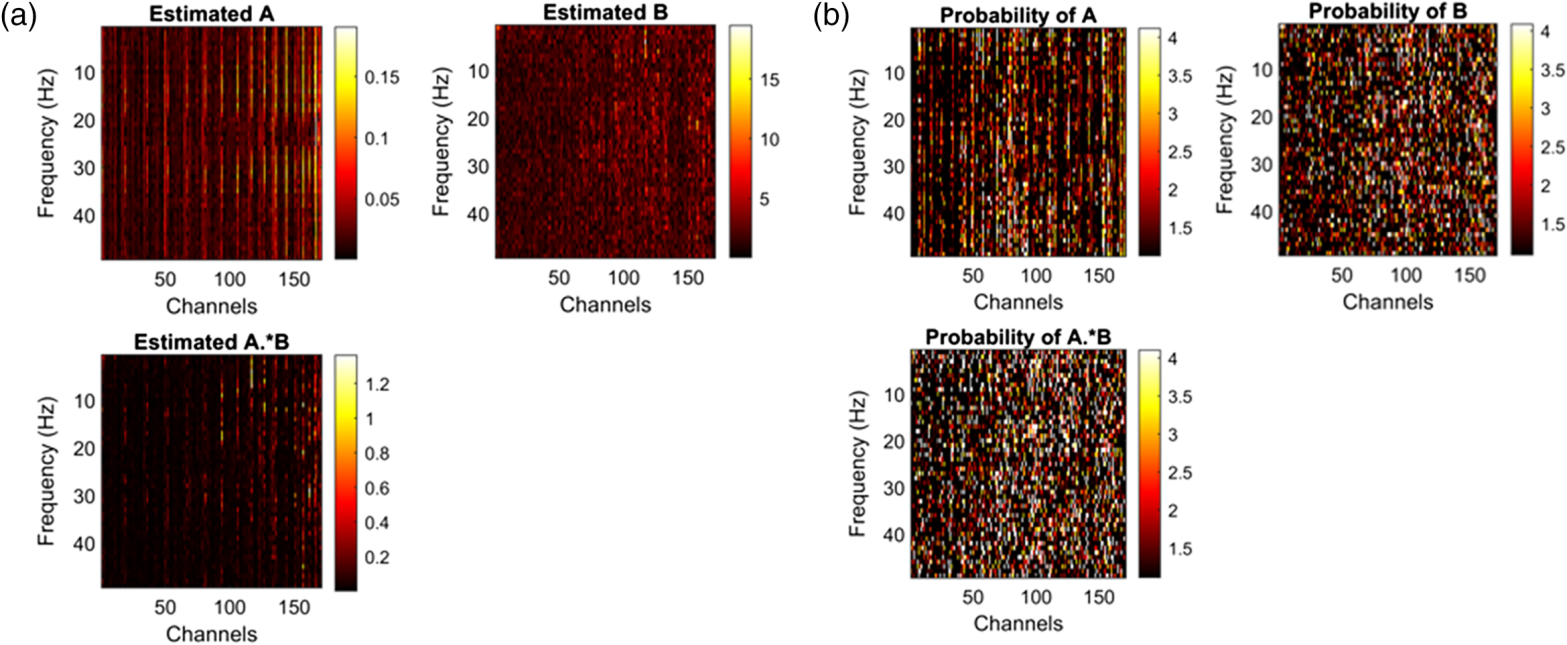
(a) Estimated coefficients for the BNS study and (b) significance of coefficients from Barbados malnutrition study for a significance level of $-\log_{10}\left(\text{threshold}\;p‐\text{value}\right)$.

Once the above step was completed, we evaluated the mediation effect based on the indirect effect A∘B. Figure 9a,b shows the estimated coefficients and their significance, respectively.

We followed a procedure similar to that used for the simulations to obtain these results. One thousand nonparametric bootstrap samples were performed. Using the Rayleigh probability distribution for complex values, we found the probabilities of each one of the elements contained in the coefficient matrices. Based on this, a threshold *p*‐value based on independence or positive dependence was calculated using FDR, and every value below or equal to that was declared significant.

Figure 9a illustrates the results for the estimated coefficients. The threshold *p*‐value for A∘B was .0159. As can be seen, the indirect effect resulted significantly, validating the differences between the control and Malnutrition groups found by Taboada‐Crispi et al. (2018) and establishing that the relationship between cognitive impairment and malnutrition has clear marks on the EEG recordings. Even further, we wanted to see which channels and frequencies reveal more about the mediation effects of the EEG cross‐spectra.

**FIGURE 10:**
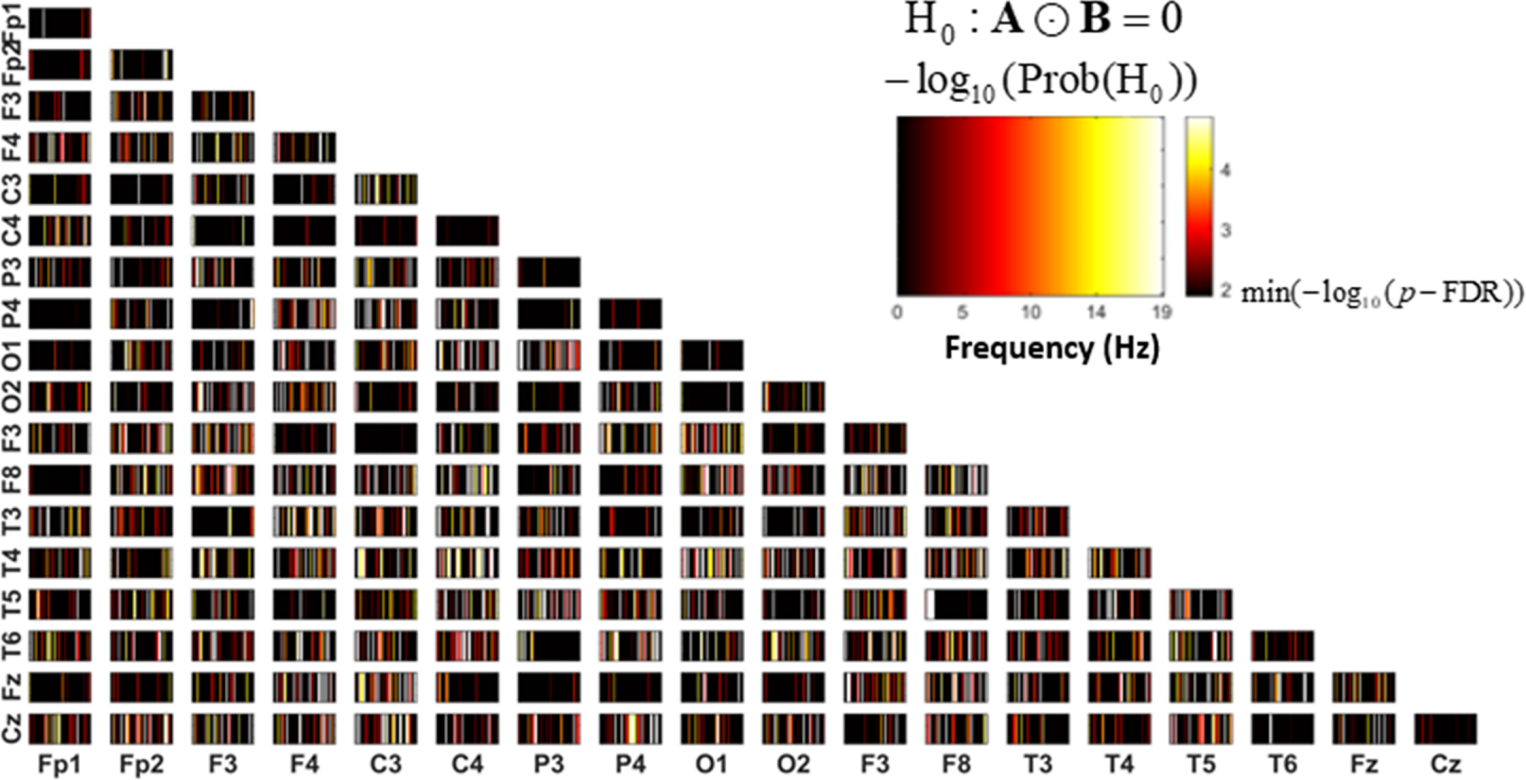
The distribution and significance of the mediation effect are seen in the combination of electrodes conforming to the 10–20 system (less the last electrode due to the average reference). Only the low diagonal is shown due to the matrix being symmetric.

Figure 10 shows *p* values for the mediation effect for all the combinations of the 18 electrodes. It needs to be remembered that we started with the classical 10–20 electrode configuration, but after the data transformation and preprocessing, one electrode was deleted. The color scale illustrates the variation of the *p* values for the 49 frequencies that form the range 0.4–19 Hz (*x*‐axis of each sub‐figure). It is shown the lower triangular due to the matrix is Hermitian. The lower bound of the color scale represents the probability of rejecting the null hypothesis for the value $-\log_{10}\left(\text{threshold}\;p‐\text{value}\right)$.

We also conducted a global mediation test for $\sum_{i}\sum_{j}a_{\mathrm{ij}}b_{\mathrm{ij}}$, with an estimated value 40.2427+15.3286i and significant *p*‐value of .0071.

### 4.3 Neurophysiological interpretation of the mediation effect in terms of the mediation frequencies

**FIGURE 11:**
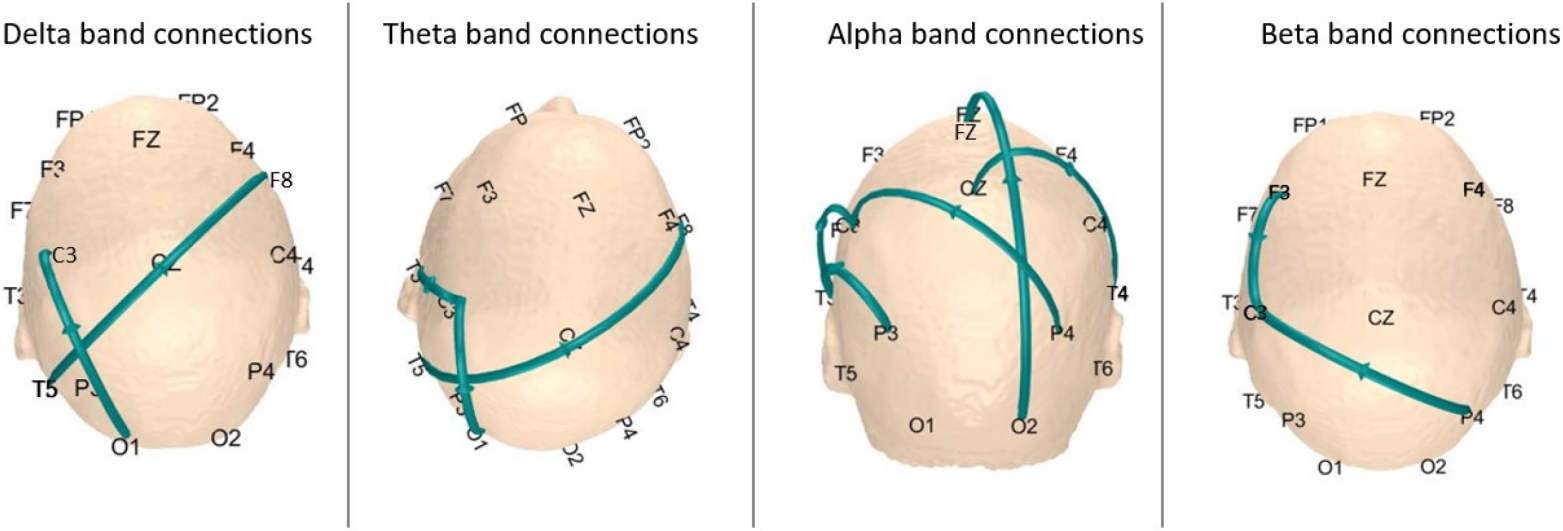
Connections in the scalp were found to be mediators between the malnutrition effect and cognition. The alpha band is the most prominent, showing 5 long (front‐occipital) and short connections. In contrast, the Beta and Delta bands show only two.

The Riemannian mediation results are expressed in pairs of frequency bands and electrodes (Table 3 and Figure 11).

**TABLE 3:** The pair of electrodes and the bins of consecutive frequencies where the mediation was statistically significant (p<.0159).

Frequency bands (Hz)	Electrodes pairs	*p* values
δ (1.9–3.9)	C3–01	.01
δ (1.9–3.9)	F8–T5	.002
θ (4–5.07)	C3–01	.02
θ (6.6–7.4)	C3–T3	.01
θ (3.9–4.6)	F8–T5	.002
α (10.9–12.5)	C3–P4	.004
α (7.4–9.3)	C3–T3	.0003
α (7–10.5)	P3–T3	.002
α (7.4–10.5)	FZ–02	.001
α (8.9–12)	Cz–T4	.0001
β (12.8–14.8)	C3–P4	.003
β (14.8–17.9)	C3–F3	.001
β (15.2–18.3)	Т4–T4	.003

*Note*: Note that for some connections, more than one band is significant. Here, we are using the IFCN classification of frequency bands: delta (1.5–3.9 Hz), low theta (4–5.4 Hz), high theta (5.8–7.4 Hz), low alpha (7.5–9.4 Hz), high alpha (9.5–12.5 Hz), low beta (12.8–14.9 Hz), and high beta (15–19.14 Hz).

We used this new method to demonstrate that both activation and coherence of the EEG spectrum mediate the effect of malnutrition during the first year of life on IQ measured at 5–11 years. Mediating connections were found in the four bands, although less in the delta, theta, and beta bands. In the alpha band, on the other hand, the connections were greater (5), of which one is a log connection between the frontal and occipital areas.

## 5 DISCUSSION

We have developed the first (to the best of our knowledge) statistical mediation model where the mediator is derived from an EEG Riemannian CST into a compressed CST with a matrix format. For this, we found a simultaneous Euclidean representation of activation and connectivity for the compressed CST. To analyze the complete frequency spectrum, we first introduced a Nuclear Norm‐Based Matrix Regression for the mediator and the outcome, and we applied this methodology to the EEG cross‐spectrum. We carried out four simulations, allowing the exposure, mediator, and outcome to depend on each other to various degrees. The proposed approach correctly identified the association in each of the simulations.

Our methodology builds upon past research on the BNS dataset. It provides a novel approach by identifying specific mediating factors: the interactions between EEG electrodes across the entire cross‐spectrum matrix, encompassing various frequencies. While prior studies solely analyzed the diagonal of this matrix (representing individual electrode activity), our approach pinpoints the precise frequencies where inter‐electrode interactions become most pronounced. This advancement unlocks a richer understanding of the brain's electrical communication pathways than previously known, obscured by the limitation of studying isolated electrode activity in previous methodologies.

Regarding the mediating effects of malnutrition on cognition, all frequency bands are affected, so communication within the same frequency and between frequencies seems dysregulated. This dysregulation has already been described in children with learning disabilities and attention difficulties; see Chabot et al. (1996) and Chabot (2001) for a review. The involvement of all EEG bands implies that early‐life malnutrition profoundly impacts the developing brain. In the delta band (1–4 Hz), the connections C3–O1 and F8–T5 suggest potential disruptions in developmental growth networks (Hu et al., 2022). Theta band alterations (4–8 Hz) in C3–O1 and C3–T3 might reflect memory and learning (Jensen & Tesche, 2002), whereas beta‐band changes (13–30 Hz) in C3–P4, C3–F3, and T4–T6 could point to alterations in active cognition and fine motor control (Canolty & Knight, 2010). Omeostasis alteration in the delta, theta, and beta bands, especially in the right hemisphere, has already been reported in children with ADHD combined (Chiarenza et al., 2018).

Another observation is that the electrode C3 is present in all frequency bands. C3 covers a large motor area also critical for cognition (Bruner, 1970). In addition, the C3–T3 connection is present in the theta and alpha bands. A connection, therefore, between motor and language processes is plausible (Piaget & Inhelder, 2008). Furthermore, the observed connections between particular electrode sites span across different brain regions, including the frontal, central (C3, Cz), parietal (P3, P4), temporal (T3, T4), and occipital (O2) lobes. These regions are associated with higher‐order cognitive functions, motor skills, and sensory and visual processing.

Two remarks regarding the connectivity patterns depicted across different frequency bands can be made. First, the majority of connections are interhemispheric. Second, these connections predominantly involve long‐distance pathways, though the absence of source connectivity complicates the interpretation. Drawing from anatomical knowledge, we understand that the frontal associative areas communicate with the parietal associative areas through the superior and inferior longitudinal fascicles (Thompson & Nelson, 2001). This communication serves to integrate information and construct a unified percept.

Additionally, memory and knowledge are represented in the cerebral cortex through widely distributed, interactive, and overlapping neuronal networks or cognits (Fuster, 1985). Cognitive functions such as perception, attention, memory (including working memory), language, and intelligence rely on neural transactions within and between these cortical memory networks (Fuster, 2022). These processes appear dysregulated due to altered brain homeostasis, which manifests across all frequency bands.

These findings highlight the potential for malnutrition to have widespread and lasting effects on brain architecture, possibly leading to altered neural connectivity, which could underlie some of the cognitive deficits observed in malnourished populations. Of note, recent studies in rodent models confirm that protein deficiency during development has a wide‐reaching impact on brain networks (Barbeito‐Andrés et al., 2018). Animals with protein malnutrition in early life rely on different networks than controls to accomplish cognitive tasks, especially those that rely on attention and executive function, changes that may represent compensatory responses in the brain following malnutrition (Rushmore et al., 2021).

Our results consistently signal the importance of the alpha band and the long‐distance connection between Occipital and Frontal electrodes, as also found by Li et al. (2022). They also align with our group's earlier work on the BNS cohort, showing how qEEG can distinguish between nutrition groups. Specifically, Taboada‐Crispi et al. (2018) demonstrated qEEG differences, including variations in theta, alpha, and beta activities across various electrode sites, indicative of delayed alpha rhythm maturation in malnourished children. Furthermore, Bringas Vega et al. (2019) identified EEG source classification as a valuable tool for assessing the impact of early malnutrition on brain function, mainly through alpha activity in the lingual gyrus.

The causal interpretation of the parameters within the compressed CST matrix mediation method developed in this article relies on robust yet untestable assumptions, particularly sequential ignorability. An example of this assumption is $\bar{y}\left(x,M\right)⊥⊥\bar{M}\left|\bar{x}\right.$, which holds if the mediators are randomly assigned to the subjects. However, in the present case, this assumption does not fit. Instead, we must assume that the mediators behave as if they were randomly assigned. This assumption cannot be empirically verified and ultimately depends on the specific context (Lindquist & Sobel, 2011). Its validity may vary across different brain regions, making it more challenging to establish causal claims. In this study, we do not consider potential confounding factors through conditioning. Despite these limitations, we still believe the proposed approach is valuable for conducting exploratory mediation analysis and identifying regions potentially mediating the relationship between treatment and outcome.

Intervening on the mediator variable without impacting the treatment variable is essential to ensure the existence of independent mechanisms influencing both the treatment and mediator, thereby excluding scenarios where the treatment and mediator necessarily co‐occur. While current technological limitations prevent precise manipulation of the mediator variable to any user‐specified value, activating or blocking brain activity is hypothetically conceivable. Also, we note that there may be potential to activate a network through direct brain stimulation without influencing the treatment variable.

In this study, we have assumed that the proposed mediation model does not include any covariates. However, it is often necessary to account for covariates in observational studies to obtain more accurate estimates. As part of our future research, we plan to expand the model and methodology to include covariates, thus enhancing the model's applicability and providing a more comprehensive analysis. Also, different distances will be evaluated and compared with the geometric one employed in this article. For instance, assessing the Wasserstein, log Cholesky, and log Euclidean distances will be interesting since they may be more efficient computationally than the Geometric distance. This approach opens the way to further explore the interaction of genes and environment according to the model of Gottlieb: Probabilistic epigenesis (Gottlieb & Lickliter, 2007).

While this study relies on sensor‐level EEG data, exploring source‐level representation using inverse solutions could offer valuable insights (Paz‐Linares et al., 2023). Unlike sensor data, the source representation can pinpoint the specific brain regions and neural networks mediating the relationship between malnutrition and cognition. By localizing this activity, we can better understand the underlying neural mechanisms crucial for designing effective interventions and treatments.

## CONFLICT OF INTEREST STATEMENT

None declared.

## Data Availability

The data that support the findings of this study are available on request from the corresponding author. The data are not publicly available due to privacy or ethical restrictions. Our code is available via GitHub (CCC‐members/Riemannian‐manifold‐mediation‐with‐an‐application‐to‐EEG‐cross‐spectra‐ (github.com)).

## APPENDIX A: HILBERT–SMITH REGULARIZATION METHOD A

### A.1

In general, datasets need regularization most of the time. This regularization is necessary due to the matricial logarithm operation involved in the mapping from the manifold to Euclidean space to ensure that the matrices have nonnegative or zero eigenvalues. To check this prerequisite, an algorithm was created that returns how many have frequencies with negative eigenvalues for a given data set of subjects. Various methods have been proposed for this, although most depend on a tuning parameter.

We choose the Hilbert–Schmidt (HS) regularization method described in Schneider‐Luftman and Walden (2015). The principal advantages are that it is a nonparametric estimation method based on theoretical inference and improves the estimation of poorly conditioned cross‐spectral matrices. The method only depends on the number of points used to find the Fourier transform of the EEG time‐domain signal. Appendix A describes in detail how to perform this operation.

Given a p‐vector‐valued (or multivariate) stationary time series $\left\{X_{t}\right\}$, where $X_{t}={\left[X_{1,t},\ldots ,X_{p,t}\right]}^{T}\in {\mathbb{R}}^{p},t\in \mathbb{Z}$ and  denotes the transpose. Let $\Sigma \left(f\right)$ indicate the cross‐spectral matrix of $\left\{X_{f}\right\}$ at frequency *f*, $\hat{\Sigma }\left(f\right)$ its estimator, and ${\hat{\Sigma }}^{*}$ the regularized spectral matrix, then the regularization is achieved by:

${\hat{\Sigma }}^{*}=\alpha_{0}\hat{\Sigma }+\beta_{0}I_{p};\;\alpha_{0},\beta_{0}>0,$where $I_{p}$ is the identity matrix of size p and the parameters $\alpha_{0}$ and $\beta_{0}$ are calculated in terms of the HS loss as follows:

$${\left(\alpha_{0},\beta_{0}\right)}_{\mathrm{HS}}={\left(\left(1-\rho_{0}\right),\eta_{0}\rho_{0}\right)}_{\mathrm{HS}},$$

$${\left(\eta_{0},\rho_{0}\right)}_{\mathrm{HS}}=\left(\frac{\tau }{p},{\left[1-\frac{K}{p}+K\frac{\tau_{1}}{\tau^{2}}\right]}^{-1}\right),$$

where $\tau =\mathrm{tr}\left\{\Sigma \right\}$, $\tau_{1}=\mathrm{tr}\left\{\Sigma^{2}\right\}$, and *K* is the number of points used to estimate $\hat{\Sigma }\left(f\right)$.

Once the matrices have been regularized, we can check again for any negative eigenvalues for cases where the regularization fails.

## APPENDIX B: DEGREES OF FREEDOM BASED ON SINGULAR VALUE THRESHOLDING B

### B.1

(B1)
$$d\hat{f}\left(\lambda \right)=\sum_{i=1}^{r}1_{\left\{b_{i}\left(\lambda \right)>0\right\}}\left(1+\sum_{1\leq j\leq p,j\neq i}\frac{\sigma_{i}\left(\sigma_{i}-\lambda \right)}{\sigma_{i}^{2}-\sigma_{j}^{2}}+\sum_{1\leq j\leq q,j\neq i}\frac{\sigma_{i}\left(\sigma_{i}-\lambda \right)}{\sigma_{i}^{2}-\sigma_{j}^{2}}\right),$$

where $\sigma \left(\hat{B}\right)=\left(b_{1}\left(\lambda \right),\ldots ,b_{r}\left(\lambda \right)\right)$ are the singular values from the nuclear‐norm‐regularized estimate $\hat{B}$ and $r=\min\left\{p,q\right\}$. When n<pq

(B2)
$$d\hat{f}\left(\tau \right)=\begin{array}{l} \sum_{i=1}^{r}1_{\left\{b_{i}\left(\tau \right)>0\right\}}(1+\frac{1}{1+\tau }\sum_{1\leq j\leq p,j\neq i}\frac{\sigma_{i}\left(1+\tau \right)\sigma_{i}-\lambda }{\sigma_{i}^{2}-\sigma_{j}^{2}}\\ +\;\frac{1}{1+\tau }\sum_{1\leq j\leq q,j\neq i}\frac{\sigma_{i}\left(1+\tau \right)\sigma_{i}-\lambda }{\sigma_{i}^{2}-\sigma_{j}^{2}}), \end{array}$$

Equations (B1) and (B2) yield an effective and fast estimate of the degrees of freedom (Zhou & Li, 2014) for both regressions based on singular value decomposition thresholding.
